# Laparoscopic versus Open Surgery for Gastric Cancer in Western Countries: A Systematic Review and Meta-Analysis of Short- and Long-Term Outcomes

**DOI:** 10.3390/jcm11133590

**Published:** 2022-06-22

**Authors:** Giovanni Maria Garbarino, Giovanni Guglielmo Laracca, Alessio Lucarini, Gianmarco Piccolino, Paolo Mercantini, Alessandro Costa, Giuseppe Tonini, Giulia Canali, Edoardo Maria Muttillo, Gianluca Costa

**Affiliations:** 1 Surgical and Medical Department of Translational Medicine, Sant’Andrea Teaching Hospital, Sapienza University of Rome, Via di Grottarossa 1035, 00189 Rome, Italy; giovanniguglielmo.laracca@uniroma1.it (G.G.L.); alessio.lucarini@uniroma1.it (A.L.); gianmarco.piccolino@uniroma1.it (G.P.); paolo.mercantini@uniroma1.it (P.M.); giulia.canali@uniroma1.it (G.C.); edardomaria.muttillo@uniroma1.it (E.M.M.); 2 UniCamillus School of Medicine, Saint Camillus International University of Health and Medical Sciences, Via di Sant’Alessandro 8, 00131 Rome, Italy; alessandro.costa@unicamillus.org; 3 Oncology Department, Fondazione Policlinico Campus Bio-Medico, University Campus Bio-Medico Hospital, Via Àlvaro del Portillo 200, 00128 Rome, Italy; g.tonini@policlinicocampus.it; 4 Surgery Center, Colorectal Surgery Unit, Fondazione Policlinico Campus Bio-Medico, University Campus Bio-Medico Hospital, Via Àlvaro del Portillo 200, 00128 Rome, Italy; g.costa@policlinicocampus.it

**Keywords:** laparoscopic gastrectomy, gastric cancer, open gastrectomy, laparoscopy, laparoscopic surgery, West, Western

## Abstract

Background. The advantages of a laparoscopic approach for the treatment of gastric cancer have already been demonstrated in Eastern Countries. This review and meta-analysis aims to merge all the western studies comparing laparoscopic (LG) versus open gastrectomies (OG) to provide pooled results and higher levels of evidence. Methods. A systematic literature search was performed in MEDLINE(PubMed), Embase, WebOfScience and Scopus for studies comparing laparoscopic versus open gastrectomy in western centers from 1980 to 2021. Results. After screening 355 articles, 34 articles with a total of 24,098 patients undergoing LG (5445) or OG (18,653) in western centers were included. Compared to open gastrectomy, laparoscopic gastrectomy has a significantly longer operation time (WMD = 47.46 min; 95% CI = 31.83–63.09; *p* < 0.001), lower blood loss (WMD = −129.32 mL; 95% CI = −188.11 to −70.53; *p* < 0.0001), lower analgesic requirement (WMD = −1.824 days; 95% CI = −2.314 to −1.334; *p* < 0.0001), faster time to first oral intake (WMD = −1.501 days; 95% CI = −2.571 to −0.431; *p* = 0.0060), shorter hospital stay (WMD = −2.335; 95% CI = −3.061 to −1.609; *p* < 0.0001), lower mortality (logOR = −0.261; 95% the −0.446 to −0.076; *p* = 0.0056) and a better 3-year overall survival (logHR 0.245; 95% CI = 0.016–0.474; *p* = 0.0360). A slight significant difference in favor of laparoscopic gastrectomy was noted for the incidence of postoperative complications (logOR = −0.202; 95% CI = −0.403 to −0.000 the = 0.0499). No statistical difference was noted based on the number of harvested lymph nodes, the rate of major postoperative complication and 5-year overall survival. Conclusions. In Western centers, laparoscopic gastrectomy has better short-term and equivalent long-term outcomes compared with the open approach, but more high-quality studies on long-term outcomes are required.

## 1. Introduction

Gastric cancer is the fifth most common cancer in the world and the third leading cause of cancer-related death. Differently to the eastern countries, in Europe no screening programs are carried out (except for a limited amount of patients affected by atrophic gastritis [1,2]), and the diagnosis often occurs in an advanced stage with a 5-year survival of around 25% [3,4]. In patients with a resectable tumor (stage IB-III) the gold standard is radical gastrectomy with D2 lymphadenectomy [5,6,7]. In Europe, for these patients, since the publication of the results of the “AIO-FLOT-4” trial, the gold standard of treatment is gastrectomy with D2 lymphadenectomy and perioperative chemotherapy [5,8].

D2 lymphadenectomy is mandatory and should be conducted by highly-experienced surgeons in high-volume centers, especially when a minimally invasive procedure is performed [9,10,11]. To date, the laparotomic approach is still the most frequently performed kind of surgery.

The first laparoscopic distal gastrectomy was described by Kitano in 1994 [12], and after that the technique gained popularity all over the world, especially in Eastern countries, where several randomized controlled trials (RCTs) on early gastric cancer (EGC) demonstrated better short-term results than open surgery, with comparable overall and disease specific survival rates [13,14,15]. Laparoscopic subtotal gastrectomy (LSG) for stage I gastric cancer (T1N0M0, T1N1M0 or T2aN0M0) was first described in 2014 by the Japanese gastric cancer treatment guidelines as one treatment option in high-volume centers [7]. Nowadays, the indications for LSG are constantly increasing, including locally advanced gastric cancer, as demonstrated by the short-term results of the Eastern countries’ multicenter RCTs [14,16,17].

Recently a non-inferiority, multicenter, international, randomized trial, performed in 13 hospitals in six European countries, showed that minimally invasive total gastrectomy after neoadjuvant therapy is not inferior regarding oncological quality of resection in comparison to open total gastrectomy in Western patients with resectable gastric cancer [18]. On the other hand. the Dutch LOGICA trial failed to demonstrate that laparoscopic gastrectomy leads to shorter hospital stay, but the oncological efficacy did not differ from that of the open gastrectomy group [19].

The differences between East and West in the overall treatment of gastric cancer have been extensively documented over the last decade; different screening protocols and endoscopic, surgical and oncological approaches are currently used in the two situations, changing the final outcomes for this pathology [20,21,22].

This systematic review and meta-analysis aims to merge all western studies comparing LG and OG available in the literature in an attempt to increase the statistical power and level of evidence supporting the use of laparoscopic gastrectomy for the treatment of gastric cancer.

## 2. Materials and Methods

### 2.1. Literature Search Strategy

A systematic review of the literature was accomplished according to the PRISMA statement [23] in order to select articles comparing laparoscopic and open surgery in the treatment of gastric cancer. In this manuscript an electronic literature search was carried out through MEDLINE (PubMed), Embase, WebOfScience and Scopus from January 1980 to 31 December 2021. The search strategy is summarized in Supplemental File S1. A manual search using other search engine, such as Google Scholar, and reference to relevant articles was also conducted. English language terms were used to perform the search, but no restrictions were adopted to exclude any paper either by language or by study type. Records retrieved were managed by Mendeley Desktop version 1.19.4. (Elselvier, Amsterdam, The Netherlands) and Covidence (Veritas Health Innovation, Melbourne, Australia).

### 2.2. Inclusion and Exclusion Criteria

PICOS criteria (population, intervention, comparison, outcomes, and study design) were used to select studies [24]. In particular, only studies reporting a comparison between laparoscopic and open approach on adult patients undergoing gastrectomy for cancer were considered. At least one peri-operative outcome of interest should be reported including overall survival (OS) and/or disease-free survival (DFS). Studies including hybrid laparoscopic-robotic procedure or comparing robotic to laparoscopic gastrectomy were excluded. Other exclusion criteria were: (1) mixed cohort of patients from Western and Eastern countries, (2) limited D1 lymphadenectomy, and (3) merged benign and malignant diseases. Papers were also excluded from the quantitative analysis if it was not possible to quantify the number of patients or the outcomes of interest, as well as case series without control group, case reports, technical notes, papers related to video, or articles with a study period of more than fifteen years. Whenever the same group of authors had presented multiple papers through the years, all the papers were considered, but only the most informative or highest quality study was included.

The work has been reported in line with PRISMA (Preferred Reporting Items for Systematic Reviews and Meta-Analyses) and AMSTAR (Assessing the Methodological quality of Systematic Reviews) Guidelines.

### 2.3. Data Extraction and Quality Assessment

According to the eligibility criteria and in order to minimize selection bias, two pairs of reviewers (GMG/GP and GGL/AL) independently reviewed each paper, assessed the quality of the studies by using the Newcastle-Ottawa Scale [25] or Jadad’s scale for RCTs [26], and even performed the data extraction. Any disagreements were discussed and resolved through a consensus meeting with a third pair of reviewers (GC/PM). The following demographic information were selected and collected if available: age, gender distribution, body mass index (BMI), ASA classification, and tumor size and/or staging. The following surgical outcomes were considered: operating time, blood loss, lymph node yield, intraoperative complications, conversion to open approach, length of hospital stay (LOS), time to first flatus, time to oral intake, duration of analgesic requirement, 30-days postoperative morbidity, and mortality, and long-term oncological outcomes (3 and 5-year OS). Whenever possible, we reported intraoperative and/or postoperative complications both as quantitative and qualitative.

### 2.4. Statistical Analysis

We analyzed continuous variables through the weighted mean difference (WMD) and 95% confidence interval (CI). For categorical variables, analysis was performed by using the odds ratio (OR) and 95% CI. Variables were converted to mean and standard deviation (SD) if reported otherwise, according to Hozo [27]. Hazard ratios (HRs) were used to analyze time to event outcomes (OS and DFS). When the HRs and 95% CI were not provided in the studies, two authors (AC and EMM), following well-established methodologies, extracted data from Kaplan-Meier (KM) curves with GraphClick software 3.0 for Mac (Arizona-Software, Phoenix, AZ, USA) and estimated the HRs using an on-line calculator (https://www.gigacalculator.com/calculators/hazard-ratio-calculator.php, accessed on 15 April 2022). The method was validated with a blind approach by correlating the data extracted from our previously published KM curves [28,29,30] with the original data or by comparing the HR of the same study reported in other meta-analyses [31]. The HR was converted to logHR and SE with variance. A positive logHR value (reference laparoscopic approach) indicated a survival benefit favoring laparoscopy over open surgery. Subgroup analyses were performed considering either the type of resection or 5-year periods. The degrees of heterogeneity between the studies were assessed by the I^2^ value. We considered an I^2^ value of 40% or lower as trivial or not important heterogeneity, and an I^2^ value of 75% or higher as considerable heterogeneity. When I^2^ value was higher than 50%, pooled estimates were obtained using a random effects model. As regards *p* value of Q index (chi-square test of heterogeneity), a *p* < 0.10 was considered significant, otherwise a conventional level of *p* < 0.05 was accepted as statistically significant. Publication bias assessment was performed by analyzing funnel plot asymmetry with Egger’s test for continuous outcomes and with Harbord’s and Peters’ test for binary outcome [32,33,34]. Statistical analysis was carried out using StataCorp2019 STATA Statistical Software: release 16 (College Station, TX, USA: StataCorp LLC).

## 3. Results

This section may be divided by subheadings. It should provide a concise and precise description of the experimental results, their interpretation, and the experimental conclusions that can be drawn.

**Figure 1:**
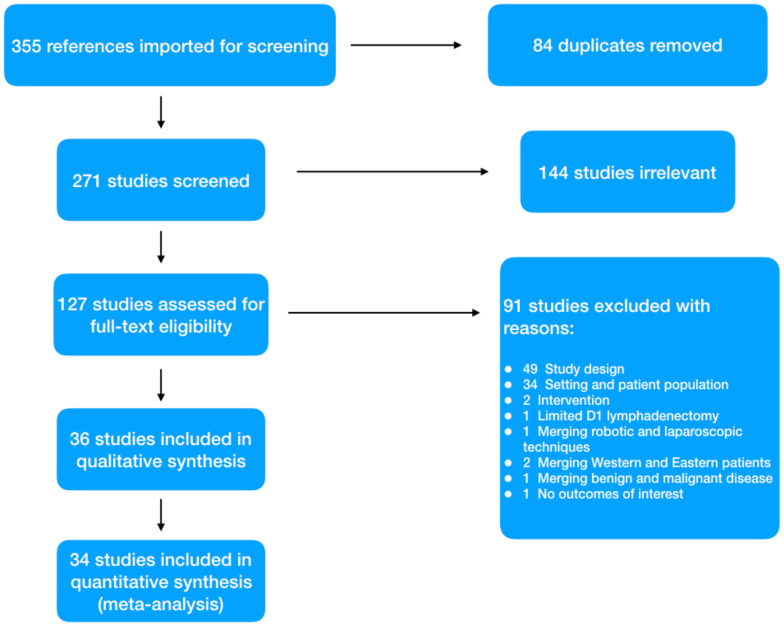
PRISMA flowchart.

Using the described search strategy, 355 items were identified. After removing duplicates and screening titles and abstract, 127 full text papers were evaluated. Ninety-one papers were further eliminated with reasons; thus 36 studies were considered eligible (Figure 1). Two studies were included only in the qualitative analysis. One retrospective case-matched study conducted in Slovenia between 1992 and 2019 has been excluded because the time study period of 27 years was considered too long to compare a technically evolving surgical approach such as laparoscopy [35]. The second, from the group of Norero et al., has been excluded because a previous case-matched study from the same authors, with a higher quality assessment score, was included [36]. Finally, 34 relevant studies were selected for the meta-analysis [18,19,28,31,37,38,39,40,41,42,43,44,45,46,47,48,49,50,51,52,53,54,55,56,57,58,59,60,61,62,63,64,65,66].

With regard to the retrieved studies, eight of these were conducted in Italy, five in the United Kingdom, four in the USA and in the Netherlands, two in France, in Germany and in Brazil, and one in Belgium, Portugal, Canada, Sweden, Turkey, Jordan, and Chile. The vast majority (17) were retrospective comparative analyses, 14 matched (eight retrospective and six prospective) and three randomized trials. All studies recruited patients between 1997 and 2019, and papers were published between 2003 and 2021. The overall quality of studies was deemed as acceptable (Newcastle-Ottawa Scale for cohort studies mean 7.7 (range 6–9) and Jadad scale for RCT mean score 3.3 (range 2–4)).

The total number of patients included in our meta-analysis was 24,098 (Open Group = 18,653; Laparoscopic Group = 5445). Baseline characteristics of the included studies are reported in Table 1.

The age was reported in all the studies except six [37,39,52,56,58,65] and the mean age in LG and OG group was 69.03 ± 4.38 years and 67.96 ± 4.09 years, respectively.

### 3.1. Comparison of Operative and Pathological Outcomes

In the laparoscopic groups conversion was reported in 20 studies [18,19,28,38,39,42,43,46,48,50,51,54,56,58,59,61,62,63,65,66] with a total of 79 conversions from laparoscopy to open surgery.

**Figure 2:**
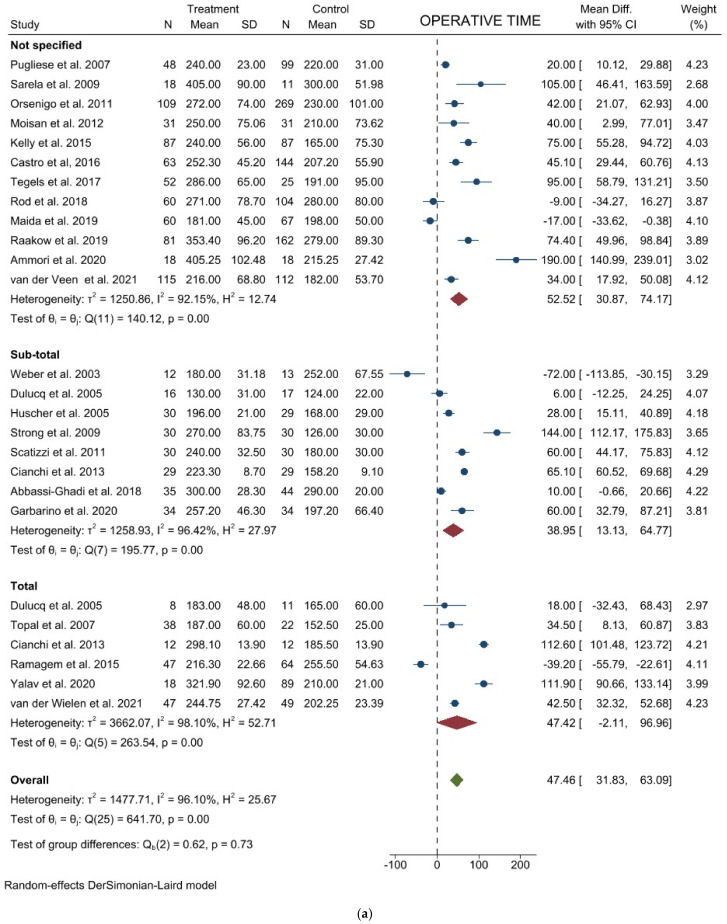

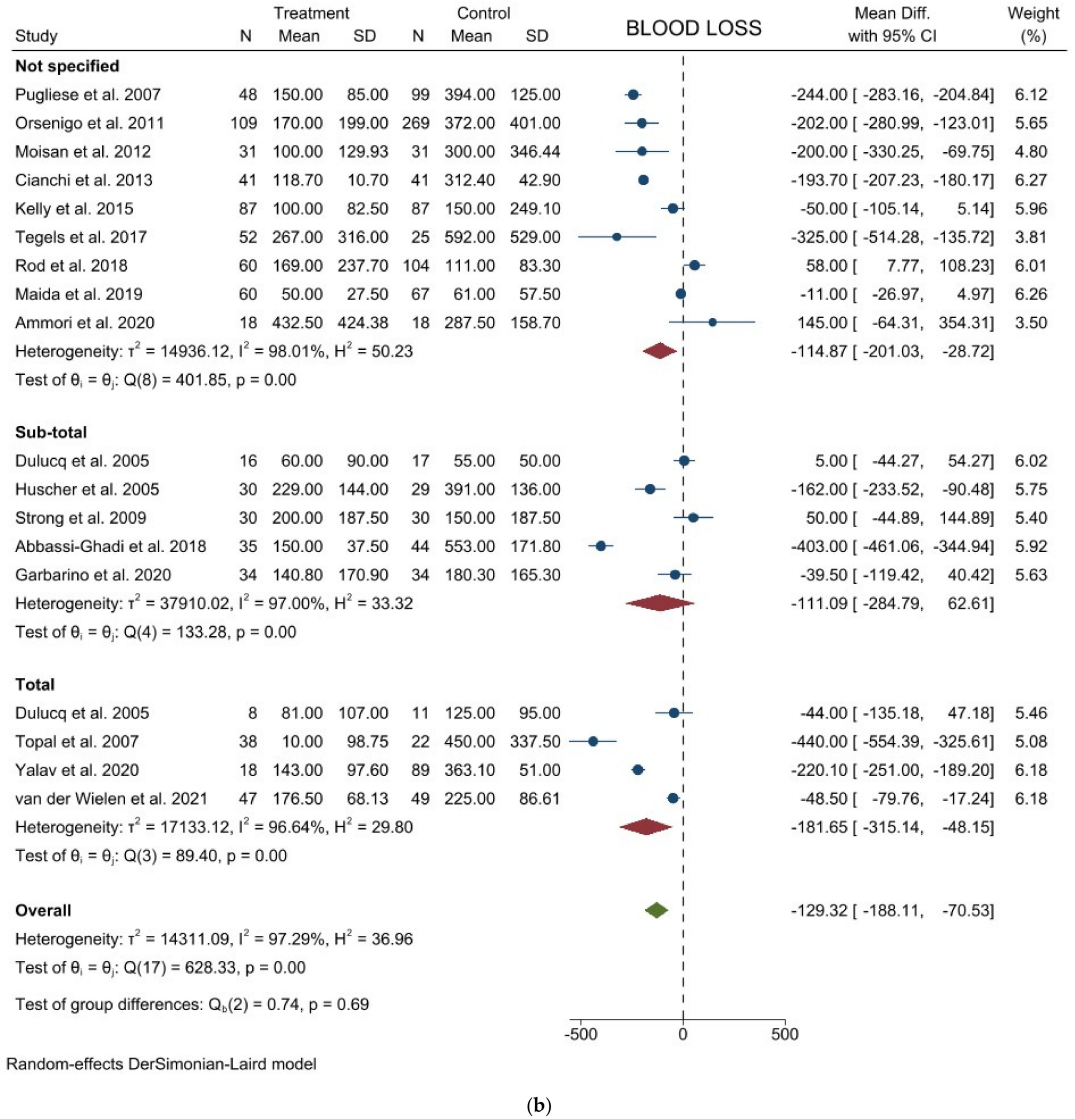

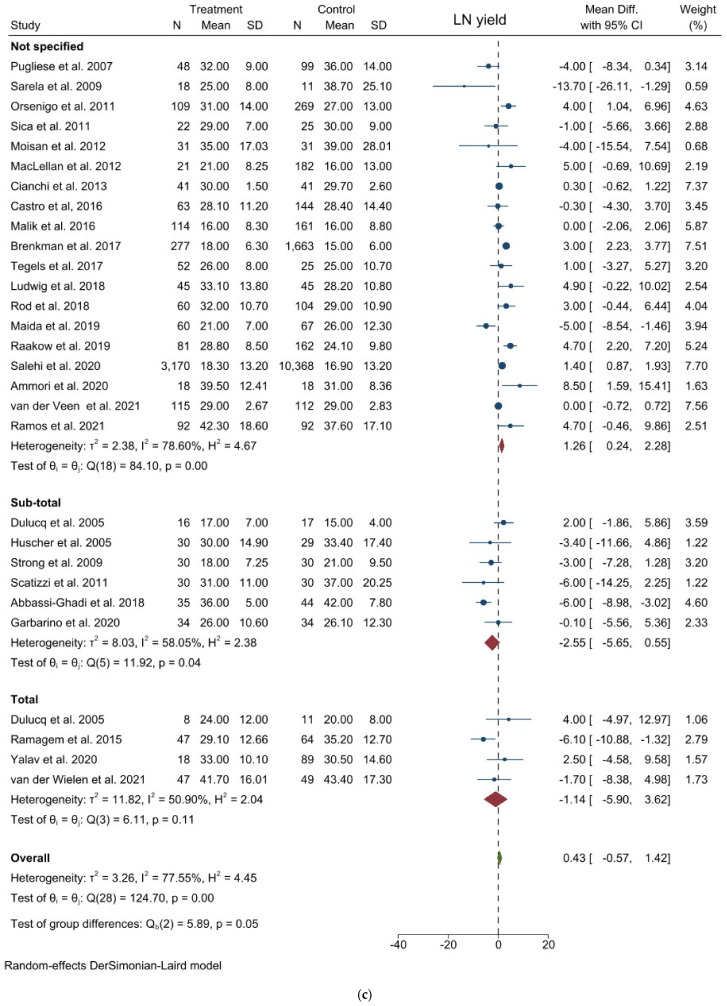
Forest plots of (**a**) Operative Time; (**b**) Blood Loss; (**c**) Lymph Node Yield.

**Figure 3:**
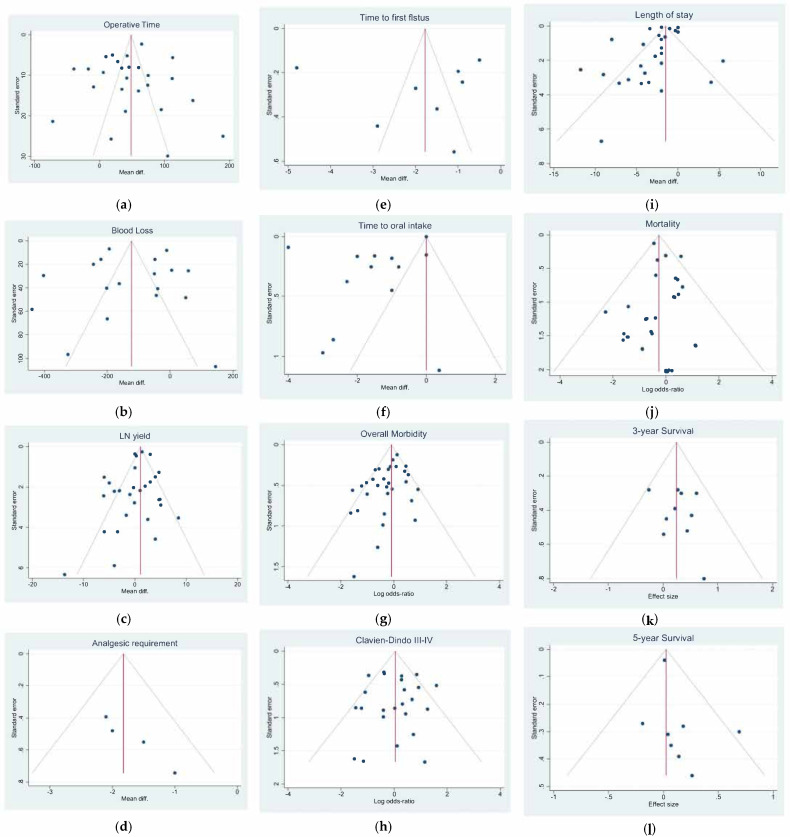
Funnel plots of (**a**) Operative time; (**b**) Blood Loss; (**c**) Lymph Node Yield; (**d**) Analgesic Requirement; (**e**) Time to First Flatus; (**f**) Time to First Oral Intake; (**g**) Overall Morbidity; (**h**) Major Complications; (**i**) Length of Stay; (**j**) Mortality; (**k**) 3-year Overall Survival; (**l**) 5-year Overall Survival.

Operative time: Twenty-four studies with 2730 patients reported the operative time. The pooled analysis demonstrated a difference in favor of the open surgery group (WMD = 47.46 min; 95% CI = 31.83–63.09; *p* < 0.001). Heterogeneity among the studies was very considerable (I^2^ = 96.10%; *p* < 0.0001), thus a random-effect model was used. No difference was noted in the subgroup analysis (Figure 2a). Egger’s test for funnel plot asymmetry showed Y Intercept at 1.46 (*p* = 0.1441) (Figure 3a).

Blood loss: Seventeen studies with 1828 patients compared the blood loss. The results showed that the blood loss amount was lower in the laparoscopic approach (WMD = −129.32 mL; 95% CI = −188.11 to −70.53; *p* < 0.0001). Heterogeneity among the studies was very considerable (I^2^ = 97.29%; *p* < 0.0001); a random-effect model was used. No difference was noted in the subgroup analysis (Figure 2b). Egger’s test for funnel plot asymmetry showed Y Intercept at −0.19 (*p* = 0.8478) (Figure 3b).

LN yield: Twenty-eight studies reported the number of harvested nodes allowing a pooled analysis of 18748 patients. The results showed that the total LNH between the two groups was similar (WMD = 0.426; 95% CI = −0.566 to 1.419; *p* = 0.3998). Heterogeneity among the studies was substantial (I^2^ = 77.55%; *p* < 0.0001), thus a random-effect model was used. A slight difference was noted in the subgroup analysis (*p* = 0.053) (Figure 2c). Egger’s test for funnel plot asymmetry showed Y Intercept at −1.37 (*p* = 0.1701) (Figure 3c).

### 3.2. Comparison of Postoperative Outcomes

Quantitative description of postoperative complications is reported in Table 2. The 30-days mortality was reported in all the studies except four [31,53,61,64] with a total mortality of 1233, 140 in the LG group and 1093 in the OG group respectively.

**Figure 4:**
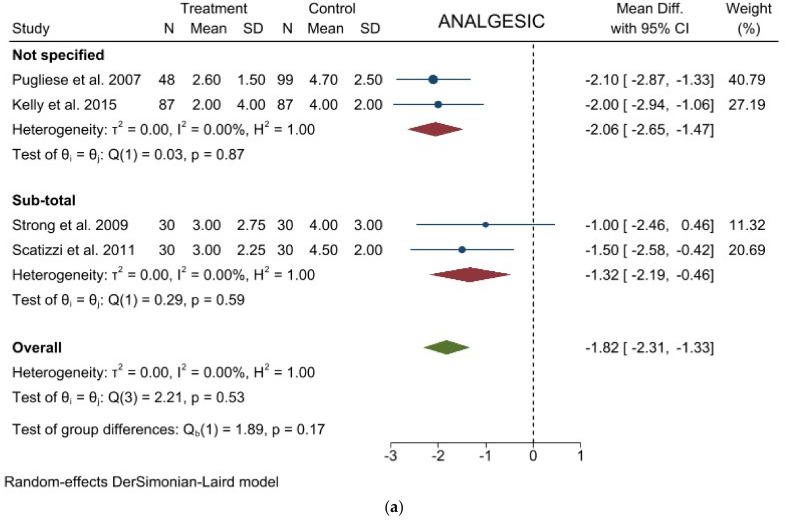

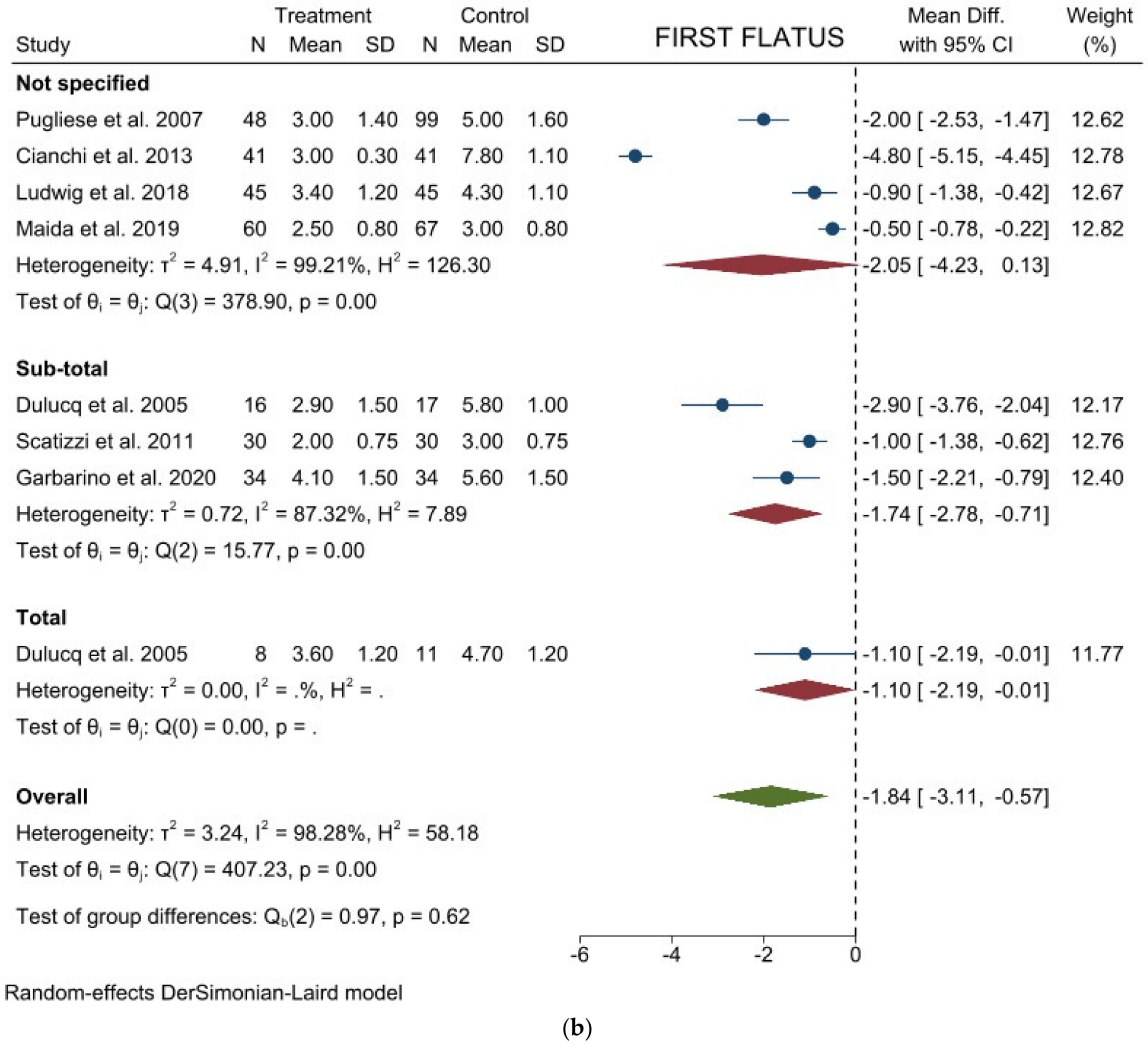

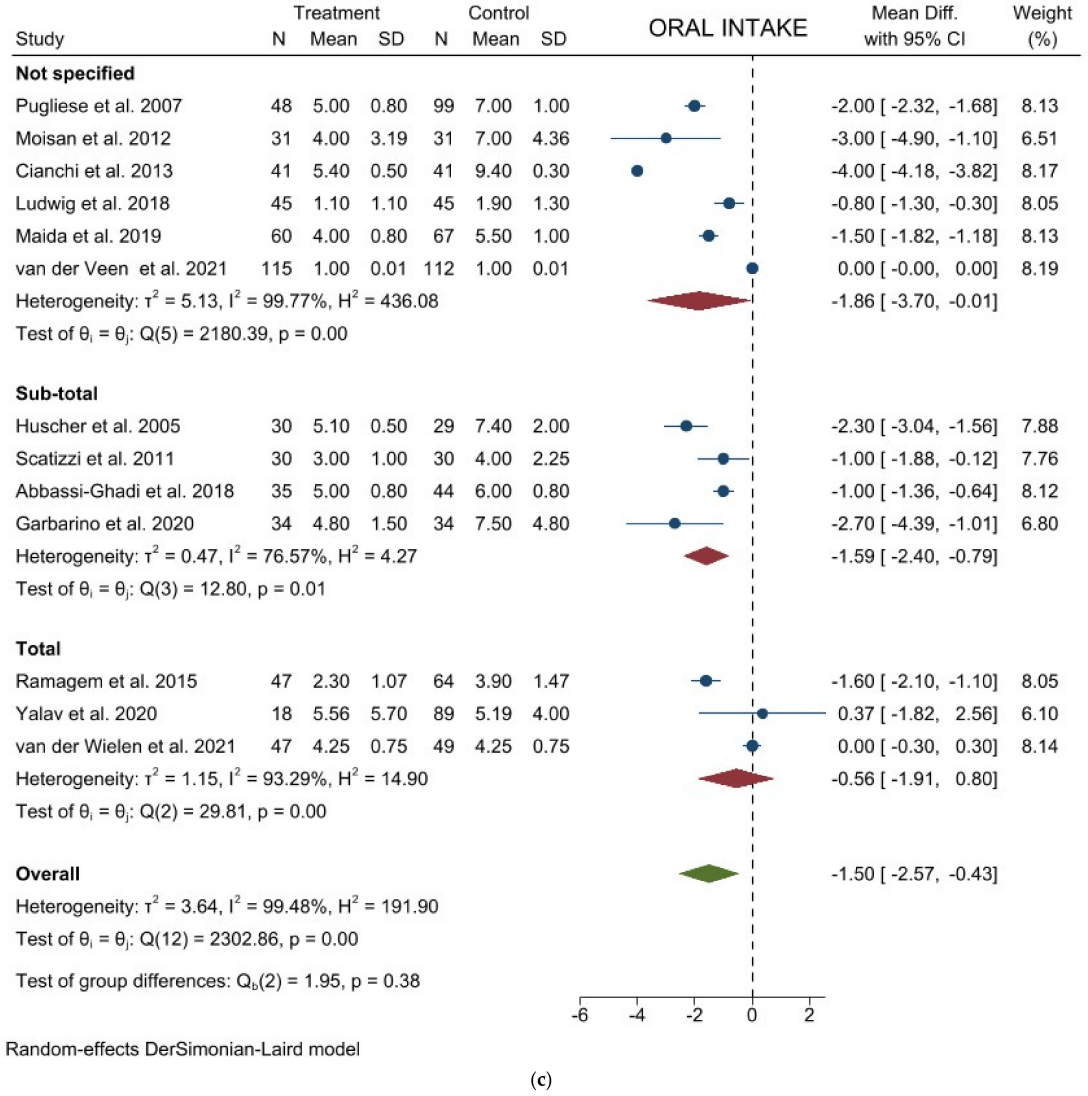

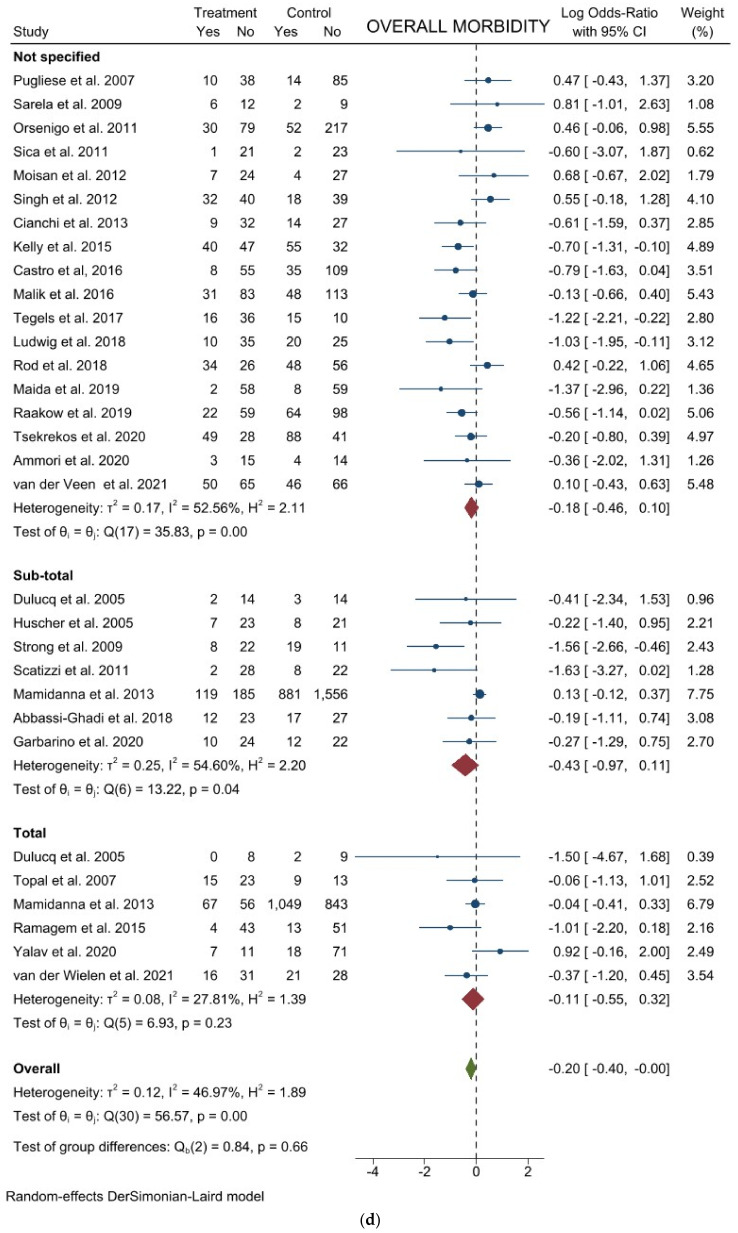

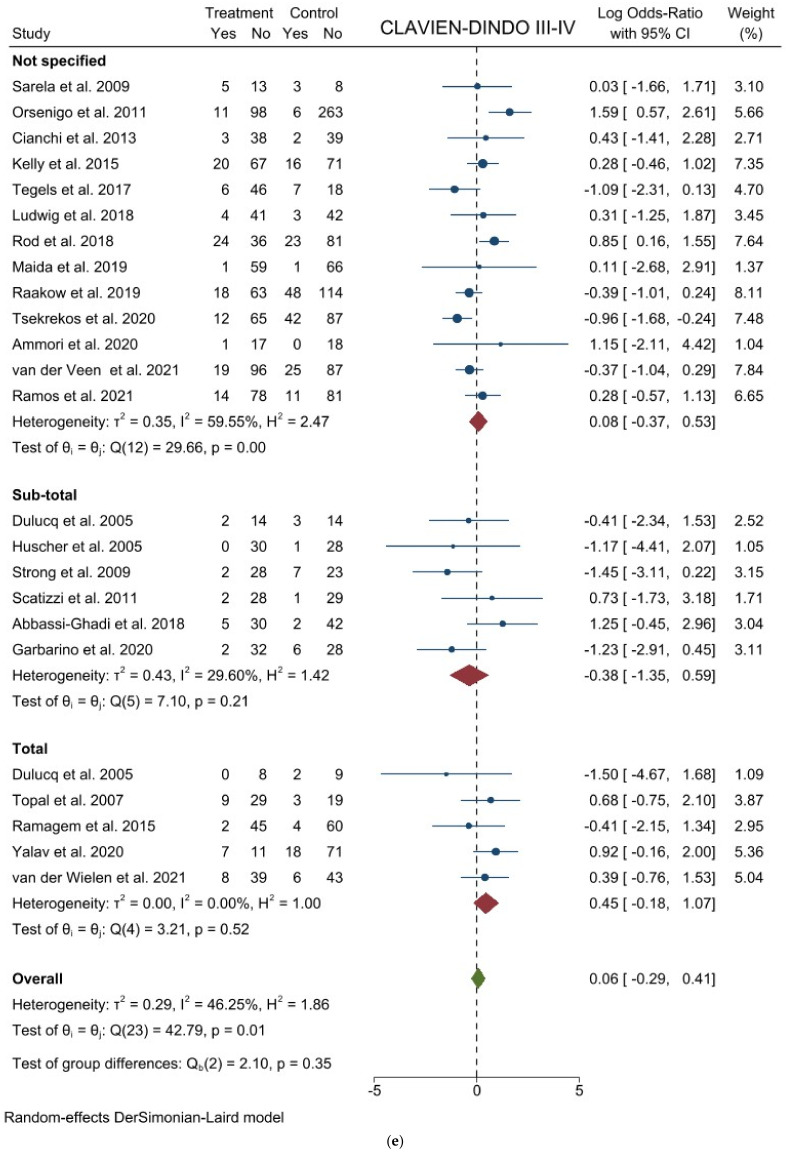

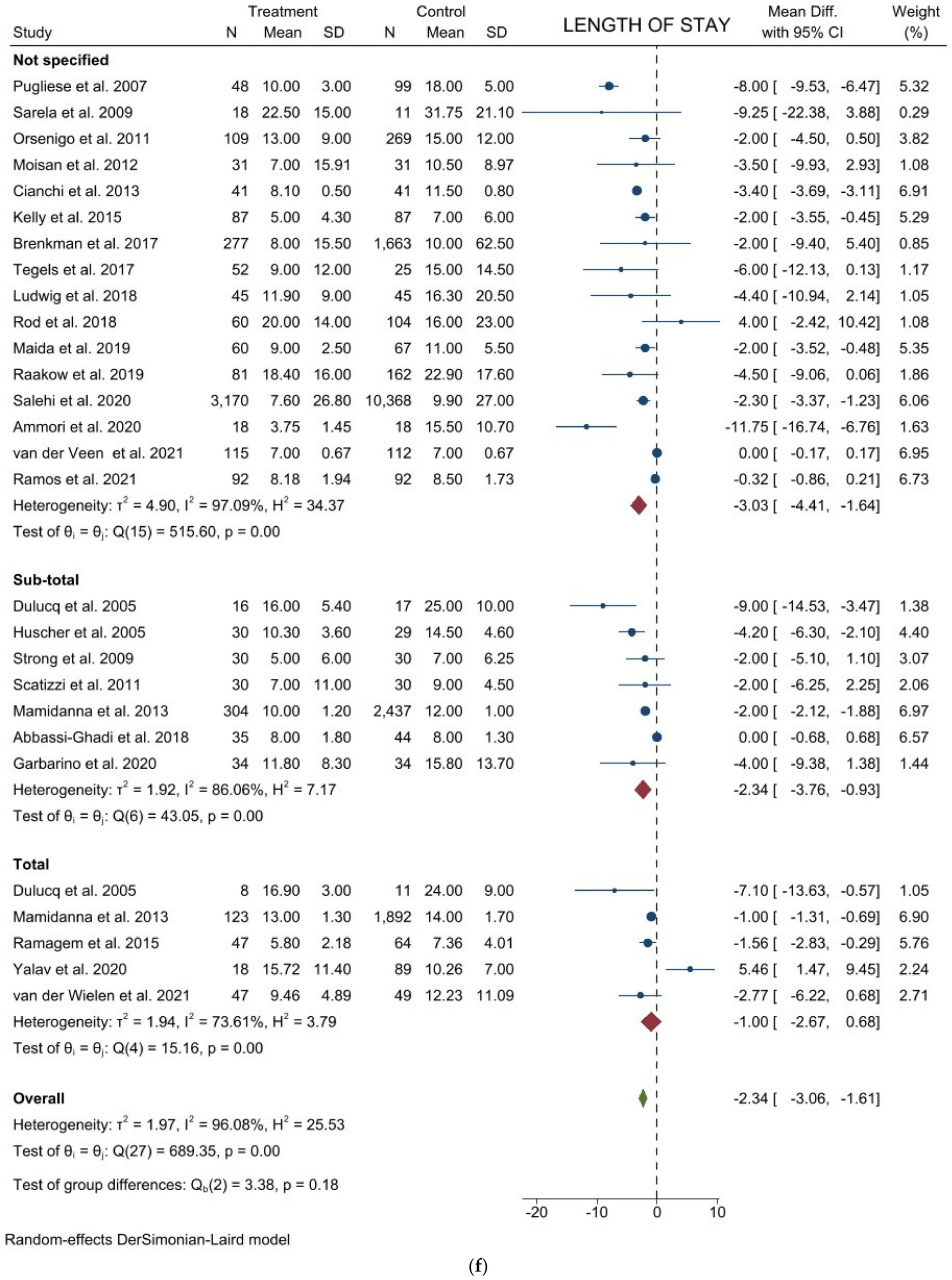

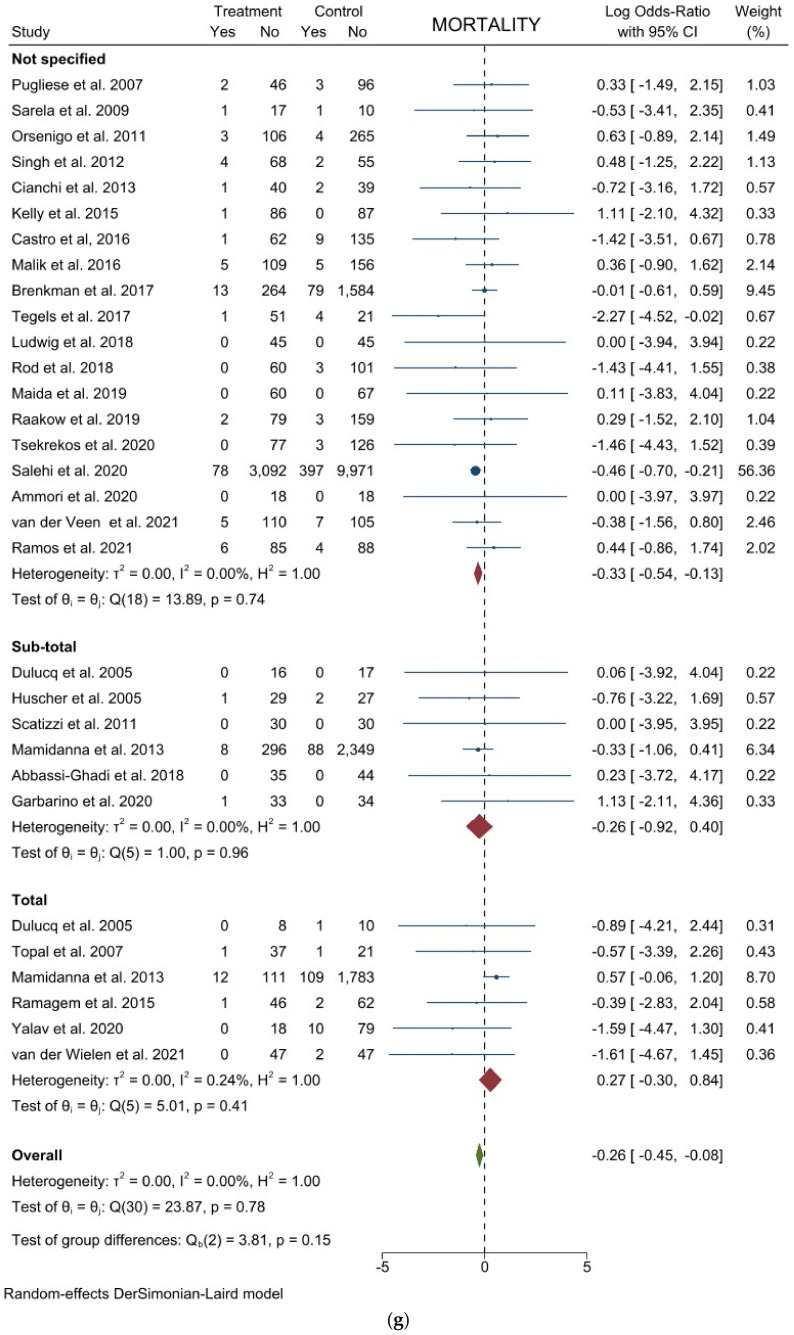
Forest plots of (**a**) Analgesic Requirement; (**b**) Time to First Flatus; (**c**) Time to First Oral Intake; (**d**) Overall Morbidity; (**e**) Major Complications; (**f**) Length of Stay; (**g**) Mortality.

Analgesic requirement: Four studies with 441 patients reported this item. The results showed a significant lower mean time of analgesic administration in laparoscopic group (WMD = −1.824 days; 95% CI = −2.314 to −1.334; *p* < 0.0001). No Heterogeneity among the studies was detected (I^2^ = 0.00; *p* = 0.5301). No difference was noted in the subgroup analysis. (Figure 4a). Egger’s test for funnel plot asymmetry showed Y Intercept at 1.43 (*p* = 0.1518) (Figure 3d).

Time to first flatus: Seven studies with 626 patients focused on this item. The results showed a significant lower mean time to first flatus in laparoscopic group (WMD = −1.840 days; 95% CI = −3.107 to −0.573; *p* = 0.0044). Heterogeneity among the studies was very considerable (I^2^ = 98.28%; *p* < 0.001). No difference was noted in the subgroup analysis (Figure 4b). Egger’s test for funnel plot asymmetry showed Y Intercept at 0.09 (*p* = 0.9272) (Figure 3e).

Time to oral intake: Thirteen studies with 1315 patients focused on this item. The results showed a significant lower mean time to first flatus in laparoscopic group (WMD = −1.501 days; 95% CI = −2.571 to −0.431; *p* = 0.0060). Heterogeneity among the studies was very considerable (I^2^ = 99.48%; *p* < 0.0001). No difference was noted in the subgroup analysis (Figure 4c). Egger’s test for funnel plot asymmetry showed Y Intercept at −0.16 (*p* = 0.8727) (Figure 3f).

Overall morbidity: From 29 studies, 8208 participants were enrolled to assess postoperative complications between the two groups. The results showed a slight statistically significant difference in postoperative complications favoring laparoscopy (logOR = −0.202; 95% CI = −0.403 to −0.000 the = 0.0499). Heterogeneity existed among the studies (I^2^ =46.97%; *p* = 0.0023). No difference was noted in the subgroup analysis (Figure 4d). Harbord’s test for funnel plot asymmetry showed Y Intercept at −1.66 (*p* = 0.0964), while Peters’ z was −1.52 (*p* = 0.1274) (Figure 3g).

Major complications (Clavien-Dindo III-IV): From 23 studies, 2769 participants were enrolled to assess major postoperative complications. The results showed no difference between the two groups (logOR = 0.058; 95% CI = −0.292 to 0.408; *p* = 0.7451). Heterogeneity existed among the studies (I^2^ = 46.25%; *p* = 0.0073). No difference was noted in the subgroup analysis (Figure 4e). Harbord’s test for funnel plot asymmetry showed Y Intercept at −0.23 (*p* = 0.8155), while Peters’ z was −0.92 (*p* = 0.3553) (Figure 3h).

Length of stay: Twenty-six studies with 22946 patients were analyzed to compare postoperative hospital stay between laparoscopic and open groups. The results showed a statistically significant difference in LOS favoring laparoscopy (WMD = −2.335; 95% CI = −3.061 to −1.609; *p* < 0.0001). Heterogeneity among the studies was very considerable (I^2^ = 96.08%; *p* < 0.0001). No difference was noted in the subgroup analysis (Figure 4f). Egger’s test for funnel plot asymmetry showed Y Intercept at −2.38 (*p* = 0.0173) (Figure 3i).

Mortality: From 29 studies, 23,701 participants were enrolled to assess postoperative mortality. The results showed a statistically significant lower risk of death in the laparoscopic cohort (logOR = −0.261; 95% the −0.446 to −0.076; *p* = 0.0056). No heterogeneity existed among the studies (I^2^ = 0.00%; *p* = 0.7778). No difference was noted in the subgroup analysis (Figure 4g). Harbord’s test for funnel plot asymmetry showed Y Intercept at −0.62 (*p* = 0.5320), while Peters’ z was −0.30 (*p* = 0.7677) (Figure 3l).

### 3.3. Comparison of Long-Term Outcomes

**Figure 5:**
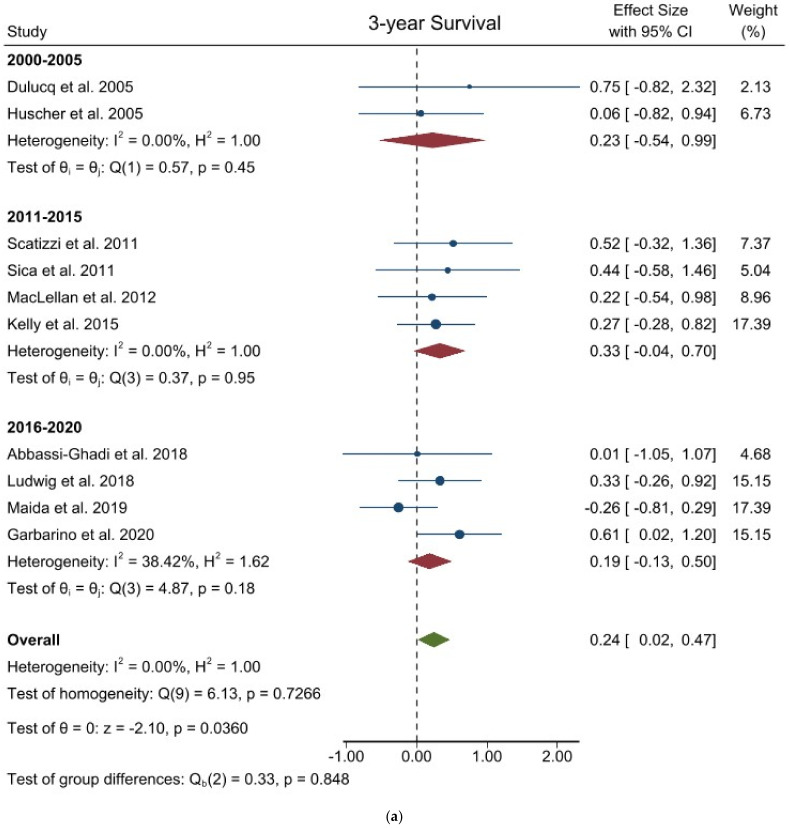

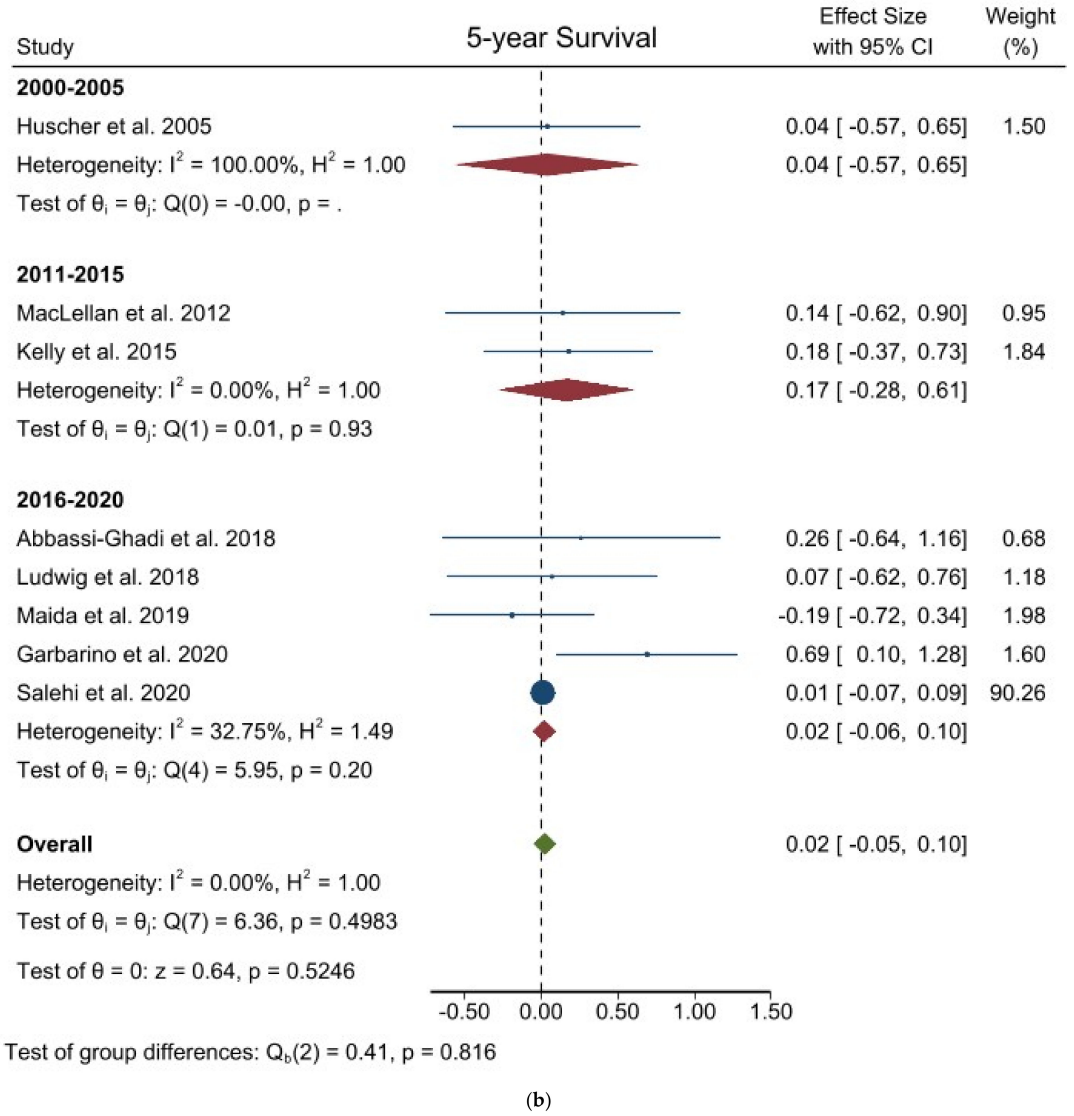
Forest plots of (**a**) 3-year Overall Survival; (**b**) 5-year Overall Survival.

Three-year overall survival: Ten studies involving 950 patients were identified to investigate the 3-year OS comparing laparoscopic versus open surgery. The pooled analysis of these studies showed that patients undergoing laparoscopic surgery had a slightly lower risk of death (logHR 0.245; 95% CI = 0.016–0.474; *p =* 0.0360) than patients in the open group which showed a cumulative mean HR of 1.106. No heterogeneity existed among the studies (I^2^ = 0.00%; *p* = 0.7266). No difference was noted in the subgroup analysis (Figure 5a). Egger’s test for funnel plot asymmetry showed Y Intercept at 0.58 (*p* = 0.5629) (Figure 3m).

Five-year overall survival: Kaplan-Meier curves from eight studies involving 14,338 patients were identified to extract data for the 5-year OS. The pooled analysis of these studies showed there was no difference between the two groups (logHR 0.024; 95% CI = −0.050 to 0.099; *p* = 0.5246). Mean HR for open surgery was 1.012. No heterogeneity existed among the studies (I^2^ = 0.00%; *p* = 0.4983). No difference was noted in the subgroup analysis. (Figure 5b) Egger’s test for funnel plot asymmetry showed Y Intercept at 1.19 (*p* = 0.2360) (Figure 3n).

## 4. Discussion

Laparoscopic surgery for gastric cancer has gained tremendous popularity over open gastrectomy because of better short-term outcomes. Several meta-analyses, mainly focusing on early gastric cancer, have demonstrated that patients undergoing LG had better early postoperative and comparable long-term outcomes when compared with those undergoing OG [67,68,69].

Moreover, results of eastern countries RCTs recently provided strong evidence in favor of laparoscopic gastrectomy concerning short-term outcomes even in the locally advanced setting [13,14,16].

Due to the differences in the epidemiology, with lower incidence but more advanced tumors at the clinical presentation in western countries, few reports in a non-Asian population have been published. The present study aimed to merge all western studies comparing LG and OG available in the literature in the attempt to increase the statistical power and level of evidence supporting the use of laparoscopic gastrectomy for the treatment of gastric cancer even in western settings.

The main concerns regarding the laparoscopic approach for gastric cancer have always been the number of lymph nodes harvested during the surgery, and the long-term outcomes [67,70,71,72].

Concerning the lymph-node yield, the results of the present meta-analysis reflect those published by Beyer et al. in a meta-analysis of RCTs regarding open versus laparoscopic gastrectomy with D2 lymphadenectomy for locally advanced gastric cancer [73]. This high-evidence study demonstrated the oncological equivalence of the laparoscopic approach for D2 lymphadenectomy compared to the open approach. Unfortunately, Beyer et al. in their meta-analysis of RCTs, concluded that the long-term oncological results could not be evaluated due to a lack of relevant data in four of the five included trials [73].

However, in this regard, a recent meta-analysis of high-quality nonrandomized studies mainly performed in Eastern Asia, showed that 5-year overall survival rate (HR 0.95, 95% CI 0.86 to 1.05, *p* = 0.28), disease-free survival (DFS) rate (HR 0.93, 95% CI 0.81 to 1.06, *p* = 0.27) and recurrence rate (OR 0.87, 95% CI 0.72 to 1.04, *p* = 0.13) were comparable between LG and OG [74].

Moreover, a recent retrospective multicenter analysis of Western centers focusing on the long-term outcomes following LG for advanced gastric cancer (stage II and III) showed the safety and feasibility of such a surgical approach [75].

Interestingly, our present study revealed a 3-year slightly lower risk of death for patients undergoing laparoscopic surgery, though such data was not confirmed by the 5-year overall survival analysis.

This result could be explained by the better short-term outcomes of laparoscopic gastrectomy: the lower inflammatory response to surgery together with a faster return to routine activities could reduce the time to the beginning of postoperative chemotherapy. Nonetheless, because this difference was not relevant at the 5-year analysis, any other possible issue should be investigated.

Despite the higher operative time, as already widely demonstrated, even this meta-analysis of western series confirmed the better short-term outcomes of laparoscopic gastrectomy: lower blood loss, lower time to first oral intake, lower time to first flatus, lower analgesic requirement, and lower hospital stay. This result suggests that the laparoscopic approach for gastrectomy should also be encouraged in western countries.

Postoperative morbidity and mortality are the main indicators for assessing the safety and feasibility of a surgical procedure. It is widely accepted that laparoscopic surgery for gastric cancer is safe and could have fewer complications than open surgery [70].

Our meta-analysis demonstrated an almost significant lower overall complication rate in LG versus OG group, whereas in the major complication (C.-D. III-IV) analysis, no differences emerged between groups.

Surprisingly, the mortality results showed a statistically significant lower risk of death in the laparoscopic cohort, without heterogeneity among the studies.

Whether for laparoscopy or open surgery, every patient diagnosed with gastric cancer needs to be discussed in a multi-disciplinary team meeting, which has been demonstrated to improve the outcomes for oncologic patients [76,77].

Non-oncological long-term outcomes, such as incisional hernia or adhesive bowel obstruction, were not reported by the majority of studies and therefore not included in our meta-analysis. These outcomes may be considered in favor of the laparoscopic approach when planning a gastrectomy.

Concerning the cost analysis, it is widely known that the laparoscopic technique itself implies higher costs, depending on the hospital policies, suppliers’ contracts and laparoscopic volume, but this is balanced by the shorter hospital length of stay. Adachi et al. demonstrated in a small series of patients undergoing a Billroth I gastrectomy that the reduction of hospital stay justifies the higher costs of laparoscopy [78]. In a Western scenario Tegels et al. demonstrated how the laparoscopic approach brings the burden of higher operative costs, but total costs were not significantly different due to shorter length of stay and less Intensive Care admission and length of stay in the laparoscopic group [42].

There are evident limitations in this meta-analysis. First, the majority of the included studies were retrospective, enrolling a small sample size of patients. It is well known that such papers may limit the conclusions on the efficacy of one technique over another. Consequently, the meta-analyses carried biases resulting from the nature of those studies. Second, publication bias is present, and a considerable degree of heterogeneity was observed in most of the outcomes. Although a random effect model was used, the results must be considered prudently. Third, the study period of the included articles was quite long for comparison of a technically evolving surgery such as laparoscopic gastrectomy. Finally, the survival analyses were carried out on a minority of papers because no sufficient western studies included data on those variables.

Despite those limitations, this study could offer a comprehensive view on outcomes of laparoscopic surgery in western gastric cancer patients.

In conclusion, laparoscopic gastrectomy is associated with longer operative time, but better short-term outcomes compared to the open approach.

Survival data of LG seemed comparable with those of open gastrectomies, but further prospective studies on long-term outcomes should be performed to confirm these results.

## Figures and Tables

**Table 1:** Baseline characteristics of studies included in the meta-analysis.

Author Year	Country	Study Period	Study Design	Tumor Stage	Extent of Resection	Sample Size		Age		M/F		Follow-Up (Months)		Newcastle-Ottawa Score
						LG	OG	LG	OG	LG	OG	LG	OG	
Weber et al., 2003 [53]	USA	1997–2000	Retrospective comparative	EGC, AGC	SG	12	13	67	67	ns	ns	18	18	7
Dulucq et al., 2005 [54]	France	1995–2004	Prospective comparative	EGC, AGC	SG	16	17	71	70	7/9	7/10	ns	ns	8
					TG	8	11	75	67	3/5	5/6			
Huscher et al., 2005 [55]	Italy	1992–1996	Randomized trial	EGC, AGC	SG	30	29	63.2	63.6	18/12	21/8	52.2	49.7	2 *
Pugliese et al., 2007 [56]	Italy	2000–2005	Retrospective comparative	EGC, AGC	SG, TG	48	99	ns	ns	29/19	ns	1–60	ns	9
Topal et al., 2007 [57]	Belgium	2003–2006	Retrospective comparative	EGC, AGC	TG	38	22	68	69	23/15	17/5	12	12	7
Sarela et al., 2009 [58]	UK	2005–2007	Retrospective comparative	EGC, AGC	SG	18	11	ns	ns	ns	ns	ns	Ns	8
Strong et al., 2009 [61]	USA	2005–2008	Retrospective case-matched	EGC, AGC	SG	30	30	71	73	13/17	14/16	36	36	7
Orsenigo et al., 2011 [62]	Italy	2002–2008	Retrospective comparative	EGC, AGC	SG	109	269	66.57	66.73	56/53	169/100	33	33	8
Scatizzi et al., 2011 [63]	Italy	2006–2009	Retrospective comparative	EGC, AGC	SG	30	30	70	69	16/14	14/16	18	18	8
Sica et al., 2011 [31]	Italy	2000–2004	Retrospective comparative	EGC, AGC	SG	22	25	67	68	13/9	13/12	39	38	8
Moisan et al., 2011 [59]	Chile	2003–2010	Retrospective case-matched	EGC, AGC	SG, TG	31	31	67	67	21/10	20/11	28	40	8
MacLellan et al., 2012 [64]	Canada	2000–2009	Retrospective comparative	EGC, AGC	SG, TG	21	182	61	57	15/6	113/69	21.3	ns	7
Singh et al., 2012 [65]	UK	2003–2010	Prospective comparative	ns	SG, TG	72	57	ns	ns	ns	ns	ns	ns	6
Cianchi et al., 2013 [66]	Italy	2008–2012	Prospective case-matched	EGC, AGC	SG, TG	41	41	73	74	25/16	25/16	ns	ns	6
Mamidanna et al., 2013 [37]	UK	2000–2010	Retrospective comparative	EGC, AGC	SG, TG	427	4329	ns	ns	276/204	6781/ 3502	ns	ns	8
Kelly et al., 2015 [38]	USA	2005–2013	Retrospective case-matched	EGC, AGC	SG, TG	87	87	64	64	37/50	54/33	11	31.1	6
Ramagem et al., 2015 [60]	Brazil	2009–2013	Retrospective comparative	EGC, AGC	TG	47	64	58	60	34/13	43/21	ns	ns	8
Castro et al., 2016 [39]	Portugal	2010–2014	Prospective comparative	ns	SG, TG	63	144	ns	ns	ns	ns	29	29	7
Malik et al., 2016 [40]	UK	2003–2014	Retrospective comparative	ns	SG, TG	114	161	73	72	55/56	101/48	60	60	7
Brenkman et al., 2017 [41]	Netherlands	2010–2014	Retrospective comparative	EGC, AGC	SG, TG	277	1663	68.5	68.4	173/104	1035/ 628	12	12	8
Tegels et al., 2017 [42]	Netherlands	2013–2014	Retrospective + prospective comparative	EGC, AGC	SG, TG	52	25	68	70	32/20	17/8	ns	ns	7
Abbassi-Ghadi et al., 2018 [46]	UK	2006–2016	Retrospective comparative	EGC, AGC	SG, TG	35	44	77	71	21/14	35/9	60	60	8
Ludwig et al., 2018 [43]	Germany	2013–2016	Prospective case-control	EGC, AGC	SG, TG	45	45	61.1	64.8	26/19	26/19	31	31	8
Rod et al., 2018 [44]	France	2005–2015	Retrospective comparative	EGC, AGC	SG, TG	60	104	62	65	37/23	63/41	ns	ns	8
Maida et al., 2019 [45]	Italy	2009–2013	Retrospective case-matched	EGC, AGC	SG, TG	60	67	71	67	28/32	36/31	ns	ns	8
Raakow et al., 2019 [47]	Germany	2005–2017	Retrospective case-matched	EGC, AGC	SG, TG	81	162	64.7	64.2	58/23	116/46	ns	ns	9
Garbarino et al., 2020 [28]	Italy	2009–2014	Retrospective case-matched	AGC	SG	34	34	70.9	71.1	23/11	21/13	31	31	8
Tsekrekos et al., 2020 [48]	Sweden	2010–2018	Retrospective comparative	EGC, AGC	SG, TG	77	129	69	68	47/30	77/52	ns	ns	9
Salehi et al., 2020 [49]	USA	2010–2016	Retrospective comparative	EGC, AGC	SG, TG	3170	10368	67.9	68.1	2162/ 1008	6814/ 2554	ns	ns	9
Yalav et al., 2020 [51]	Turkey	2015–2018	Retrospective comparative	EGC, AGC	TG	18	89	57.3	59.4	12/6	58/31	25	15	8
Ammori et al., 2020 [50]	Jordan	2017–2019	Retrospective case-matched	EGC, AGC	SG, TG	18	18	60.5	57.5	12/6	13/5	ns	ns	8
van der Veen et al., 2021 [19]	Netherlands	2015–2018	Randomized trial	EGC, AGC	SG, TG	115	112	67.9	66.9	68/47	72/40	12	12	4 *
Ramos et al., 2021 [52]	Brazil	2009–2019	Retrospective case-matched	EGC, AGC	SG, TG	92	92	ns	ns	50/42	49/43	31	31	8
van der Wielen et al., 2021 [18]	Netherlands	2015–2018	Randomized trial	EGC, AGC	TG	47	49	59.4	61.8	28/19	32/17	12	12	4 *

*: Jadad scale for randomized trials, EGC: Early Gastric Cancer, AGC: Advanced gastric Cancer, SG: Sub-total Gastrectomy, TG: Total Gastrectomy, ns: non specified.

**Table 2:** Table of complications of studies included in the meta-analysis.

Author Year	Total			Complications		Grading of Complications		Mortality		Readmissions		Reoperations		Duodenal Stump Leak		Anastomotic Leak	
	LG		OG	LG	OG	LG	OG	LG	OG	LG	OG	LG	OG	LG	OG	LG	OG
Weber et al., 2003 [53]	12		13	ns	ns	ns	ns	ns	ns	ns	ns	ns	ns	ns	ns	ns	ns
Dulucq et al., 2005 [54]	SG	16	17	2	3	C.D. I/II 1 C.D. III/IV 1	C.D. I/II 3 C.D. III/IV 0	0	1	ns	ns	1	0	1	0	0	0
	TG	8	11	0	1	C.D. I/II 0 C.D. III/IV 0	C.D. I/II 1 C.D. III/IV 0	0	0	ns	ns	0	0	0	0	0	0
Huscher et al., 2005 [55]	30		29	7	8	C.D. I/II 7 C.D. III/IV 0	C.D. I/II 7 C.D. III/IV 1	1	2	ns	ns	ns	ns	0	1	0	0
Pugliese et al., 2007 [56]	48		99	10	14	ns	ns	2	3	ns	ns	0	0	2	1	0	2
Topal et al., 2007 [57]	38		22	15	9	TOSGS I/II 6 TOSGS III/V 9	TOSGS I/II 6 TOSGS III/V 3	1	1	ns	ns	6	0	0	0	2	0
Sarela et al., 2009 [58]	18		11	ns	ns	ns	ns	1	1	ns	ns	3	2	3	1	1	0
Strong et al., 2009 [61]	30		30	8	13	C.D. I/II 6 C.D. III/IV 2	C.D. I/II 6 C.D. III/IV 7	ns	ns	ns	ns	ns	ns	0	0	0	1
Orsenigo et al., 2011 [62]	109		269	30	52	ns	ns	3	4	ns	ns	11	6	20	14	ns	ns
Scatizzi et al., 2011 [63]	30		30	2	8	TOSGS I/II 2 TOSGS III/V 0	TOSGS I/II 6 TOSGS III/V 2	0	0	1	1	1	1	0	1	2	0
Sica et al., 2011 [31]	22		25	ns	ns	ns	ns	ns	ns	ns	ns	ns	ns	ns	ns	ns	ns
Moisan et al., 2012 [59]	31		31	8	6	ns	ns	ns	ns	ns	ns	4	4	2	0	2	2
MacLellan et al., 2012 [64]	21		182	ns	ns	ns	ns	ns	ns	ns	ns	ns	ns	ns	ns	ns	ns
Singh et al., 2012 [65]	72		57	32	18	ns	ns	4	2	ns	ns	ns	ns	ns	ns	ns	ns
Cianchi et al., 2013 [66]	41		41	9	14	ns	ns	1	2	ns	ns	3	2	2	2	0	2
Mamidanna et al., 2013 [37]	480		10233	208	2661	ns	ns	23	571	58	1044	22	409	ns	ns	ns	ns
Kelly et al., 2015 [38]	87		87	27	42	C.D. I/II 11 C.D. III/IV 16	C.D. I/II 26 C.D. III/IV 16	1	0	ns	ns	ns	ns	6	4	4	4
Ramagen et al., 2015 [60]	47		64	4	13	ns	ns	1	2	2	3	3	4	ns	ns	1	3
Castro et al., 2016 [39]	63		144	8	35	ns	ns	1	9	ns	ns	ns	ns	ns	ns	ns	ns
Malik et al., 2016 [40]	114		161	31	48	ns	ns	5	5	ns	ns	ns	ns	ns	ns	ns	ns
Brenkman et al., 2017 [41]	277		1663	ns	ns	ns	ns	13	79	ns	ns	ns	ns	ns	ns	ns	ns
Tegels et al., 2017 [42]	52		25	16	15	C.D. I/II 10 C.D. III/IV 6	C.D. I/II 8 C.D. III/IV 7	1	1	6	4	ns	ns	ns	ns	2	10
Abbassi-Ghadi et al., 2018 [46]	35		44	35	47	C.D. I/II 30 C.D. III/IV. 5	C.D. I/II 45 C.D. III/IV 2	0	0	3	1	3	1	ns	ns	0	1
Ludwig et al., 2018 [43]	45		45	10	20	C.D. I/II 6 C.D. III/IV 4	C.D. I/II 17 C.D. III/IV 3	0	0	ns	ns	1	1	1	1	1	1
Rod et al., 2018 [44]	60		104	34	48	C.D. I/II 10 C.D. III/IV 24	C.D. I/II 25 C.D. III/IV 23	0	3	ns	ns	16	6	8	10	10	10
Maida et al., 2019 [45]	60		67	2	8	C.D. I/II 1 C.D. III/IV 1	C.D. I/II 7 C.D. III/IV 1	0	0	ns	ns	1	0	0	1	ns	0
Raakow et al., 2019 [47]	81		162	22	64	C.D. I/II 4 C.D. III/IV 18	C.D. I/II 16 C.D. III/IV 48	2	3	ns	ns	5	22	1	1	4	9
Garbarino et al., 2020 [28]	31		34	7	11	C.D. I/II 5 C.D. III/IV 2	C.D. I/II 5 C.D. III/IV 6	1	0	ns	ns	2	6	2	3	1	1
Tsekrekos et al., 2020 [48]	77		129	49	88	C.D. I/II 37 C.D. III/IV 12	C.D. I/II 46 C.D. III/IV 42	0	3	ns	ns	ns	ns	ns	ns	1	18
Salehi et al., 2020 [49]	3170		10368	ns	ns	ns	ns	78	397	205	791	ns	ns	ns	ns	ns	ns
Yalav et al., 2020 [51]	18		89	7	18	C.D. I/II ns C.D. III/IV 7	C.D. I/II ns C.D. III/IV 12	0	10	3	18	ns	ns	2	3	2	4
Ammori et al., 2020 [50]	18		18	3	4	ns	ns	0	0	0	1	1	0	ns	ns	ns	ns
Van der Veen et al., 2021 [19]	115		112	50	46	C.D. I/II 31 C.D. III/IV 14	C.D. I/II 21 C.D. III/IV 17	12	10	11	10	ns	ns	ns	ns	10	11
Ramos et al., 2021 [52]	92		92	ns	ns	C.D. I/II ns C.D. III/IV 14	C.D. I/II ns C.D. III/IV 11	6	4	ns	ns	ns	ns	ns	ns	ns	ns
Van der Wielen et al., 2021 [18]	47		49	16	21	C.D. I/II 8 C.D. III/IV 8	C.D. I/II 15 C.D. III/IV 4	0	2	ns	ns	1	2	ns	ns	4	5

## Data Availability

Not applicable.

## Supplementary Materials

The following supporting information can be downloaded at: https://www.mdpi.com/article/10.3390/jcm11133590/s1, File S1: Search strategy.
