# Supplementary material for: Laparoscopic versus Open Surgery for Gastric Cancer in Western Countries: A Systematic Review and Meta-Analysis of Short- and Long-Term Outcomes
Source: J Clin Med. 2022 Jun 22;11(13):3590. doi: 10.3390/jcm11133590 (PMC9267365; doi:10.3390/jcm11133590)
Supplement: Supplementary file 1 [file jcm-11-03590-s001.zip › jcm-1737435-supplementary.pdf]

File S1: Search strategy

Keywords

Terms connected by **OR**	**AND**	Terms connected by **OR**	**AND**	Terms connected by **OR**	**AND**	Terms connected by **OR**
“Gastric cancer” [MeSH Terms] **OR** “Gastric neoplasm “[MeSH Terms] **OR** “Gastric adenocarcinoma” **OR** “Stomach cancer” [MeSH Terms] [MeSH Terms] **OR** “Stomach neoplasm” [MeSH Terms **OR** “Stomach Adenocarcinoma” [MeSH Terms]		“Laparscopy” [MeSH Terms] **OR** “Laparoscopic” [MeSH Terms] **OR** “Minimally Invasive” [MeSH Terms]		“Gastrectomy” [MeSH Terms] **OR** “Gastric resection” [MeSH Terms]		“West” [MeSH Terms] **OR** “Western” [MeSH Terms] **OR** “Europe” [MeSH Terms] **OR** “America” [MeSH Terms] **OR “**US” [MeSH Terms]

“Humans” filter was applied as well as “Publication Date” from 01/01/1980 to 31/12/2021
